# Expanding Horizons: The Realities of CAD, the Promise of Artificial Intelligence, and Machine Learning’s Role in Breast Imaging beyond Screening Mammography

**DOI:** 10.3390/diagnostics13132133

**Published:** 2023-06-21

**Authors:** Tara A. Retson, Mohammad Eghtedari

**Affiliations:** Department of Radiology, University of California, San Diego, CA 92093, USA; meghtedari@health.ucsd.edu

**Keywords:** artificial intelligence, breast imaging, study comparison, beyond mammography

## Abstract

Artificial intelligence (AI) applications in mammography have gained significant popular attention; however, AI has the potential to revolutionize other aspects of breast imaging beyond simple lesion detection. AI has the potential to enhance risk assessment by combining conventional factors with imaging and improve lesion detection through a comparison with prior studies and considerations of symmetry. It also holds promise in ultrasound analysis and automated whole breast ultrasound, areas marked by unique challenges. AI’s potential utility also extends to administrative tasks such as MQSA compliance, scheduling, and protocoling, which can reduce the radiologists’ workload. However, adoption in breast imaging faces limitations in terms of data quality and standardization, generalizability, benchmarking performance, and integration into clinical workflows. Developing methods for radiologists to interpret AI decisions, and understanding patient perspectives to build trust in AI results, will be key future endeavors, with the ultimate aim of fostering more efficient radiology practices and better patient care.

## 1. Introduction

In the rapidly evolving field of medical imaging, artificial intelligence (AI) has emerged as a powerful tool with the potential to revolutionize diagnosis, quantitative tasks, and numerous aspects of clinical practice. Breast imaging has always been at the forefront of embracing and incorporating technological advances in radiology, and AI is no exception. The AI applications for mammography have been widely explored in both the research and commercial realms, resulting, at times, in public fanfare. For example, in 2020 a paper by McKinney et al., backed in part by Google, propelled breast imaging AI into the public spotlight, as it showed that an AI algorithm could outperform radiologists in predicting breast cancer on screening mammography [1]. Although most research and product development in breast imaging have focused on cancer detection or density assessment on screening mammography, a radiologist’s responsibilities extend beyond these tasks to include various modalities and clinical and administrative duties. This review aims to take a deeper look into the diversity of applications of AI towards breast imaging, including ultrasound evaluation, comparison with prior studies, and the fusion of clinical and imaging information for both diagnosis and risk assessment. In addition, we highlight how AI can facilitate administrative tasks such as compliance with the Mammography Quality Standards Act (MQSA), clinical scheduling, and protocoling, with the goal of easing the non-interpretive workload of radiologists. This multifaceted subject is also not without a necessary discussion of the limitations and challenges faced by AI implementation in this sphere. By examining these diverse facets of AI applications in breast imaging, we aim to provide an overview that will aid both clinicians and researchers in understanding the current landscape and potential future directions in this field, as well as highlight how these AI-driven processes can augment both the accuracy and efficiency of technologists and radiologists, leading to improved patient care.

## 2. How Did We Get Here?

### 2.1. The Realities of Computer-Aided Detection

As we discuss the scope of AI breast imaging applications, it is important to appreciate the historical context that led to the current landscape. Breast imagers have always been connected with cutting-edge efforts to use technology for the enhancement of cancer detection. A notable milestone along this pathway was when the Food and Drug Administration (FDA) approved the first computer-aided detection (CAD) software for screening mammography in 1998, the R2 Technology ImageChecker M1000 [2]. This technology ushered in a new era in the field. The ImageChecker M1000 worked by using a laser digitizer to convert mammography films into 6k × 6k pixel grey-scale digital images, which were then processed by a machine learning algorithm operating on a PC-based processor. This early machine learning architecture comprised two parallel processing algorithms specifically designed to detect (1) calcifications (defined as a minimum of three bright spots reaching a predefined threshold), and (2) masses (objects larger than calcifications with centers of radiating lines and borders defined by gradient changes). The network was trained on only several hundred cases of known breast cancer and normal mammograms, encompassing a mix of calcifications and masses. The ImageChecker M1000 needed approximately six minutes to analyze the four views of a standard screening mammogram [2,3]. Following the approval for CAD reimbursement in 2002, its use in screening mammography rapidly became standard, with 83% of practices using CAD by 2012 [4]. 

Despite early excitement surrounding CAD algorithms, with some suggesting that CAD would outperform radiologists or act as a second reader for screening studies, enthusiasm diminished as the limitations of CAD emerged. A critical concern with traditional CAD was its high false-positive rates, leading to increased diagnostic studies [5], which, in turn, resulted in unnecessary further testing and patient anxiety. Further, CAD systems frequently provided results without the ability to explain how they arrived at those conclusions, and without offering a quantitative measure of suspicion, making it opaque and difficult for clinicians to understand or trust the results. This likely contributed to limited efficacy and a lack of radiologist engagement, with a study by Mahoney showing that radiologists found CAD markings easy to dismiss in up to 88% of cases [6]. Later studies also showed that CAD did not actually impact cancer detection rates and that practicing radiologists rarely altered their opinions based on CAD [7]. 

### 2.2. The Promises of AI and Its Advancements over Traditional CAD

Nearly 25 years after the initial CAD applications, a new generation of AI-based computer-aided detection is revolutionizing the field of radiology and breast imaging in particular. Conventional CAD approaches to breast cancer identification rely on a software programmer to define specific and detailed rules and instructions to perform a task. In contrast, several types of artificial intelligence called machine learning (ML) or deep learning (DL) employ numerous examples, often numbering in the hundreds or thousands to “learn” how to perform a task without explicit instructions. For instance, consider the task of marking suspicious areas of calcifications on a mammogram. In conventional programming, one must define the exact imaging features of calcifications in terms of shape and pixel intensity to enable the program to identify pixels of calcification versus those of normal tissue. The accuracy of such a program depends on the programmer’s ability to define and describe the imaging features. In contrast, the ML approach does not require definitions of suspicious or normal features to be pre-defined in the coding. Instead, the programmer writes an algorithm to analyze examples of normal tissue and examples of calcifications, allowing the algorithm to determine which features it will use to differentiate the two. Although ML techniques were first developed in the 1980s, they experienced a dramatic recent increase in popularity due to several major and related changes in computer science. Foremost, computing power has become exponentially more affordable and accessible over the past decade. This has enabled algorithm architectures to grow more complex, in turn, allowing them to take on increasingly nuanced problems. Concurrently, the increased computing has facilitated the use of substantially larger data sets, with ML algorithms now being trained on thousands or tens of thousands of images. By expanding the breadth and quantity of training images an algorithm can learn to make increasingly refined distinctions, much like a radiology resident improves by seeing a multitude of cases during their training. In this vein of continuous improvement the proficiency of AI has drastically exceeded the performance of traditional CAD techniques, approaching or exceeding the accuracy of radiologists at various tasks in mammography including lesion detection [1,8,9]. AI is also gaining increasing traction for a role as a digital second reader [10,11], with some studies proposing the use of AI to sort confidently negative studies for worklist reduction [12,13]. 

**Figure 1:**
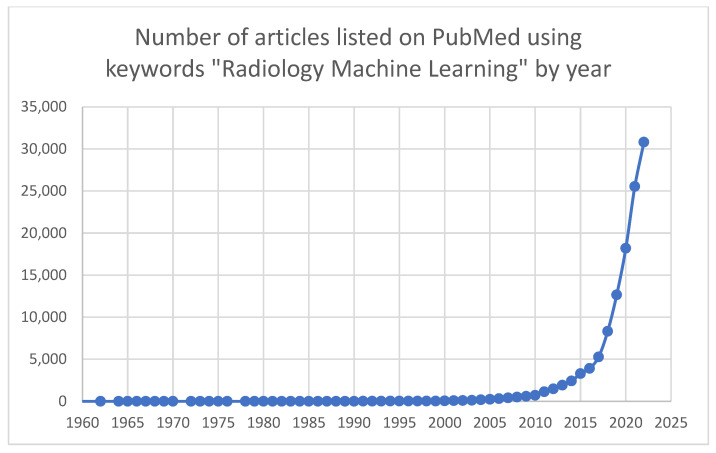
Graph showing the number of articles per year listed on PubMed when using the search keywords “radiology machine learning”.

The total number of ML research projects in radiology has increased exponentially over the last decade. When searching PubMed using the keywords “Radiology Machine Learning”, there were 713 results in 2010, compared to 30,814 results in 2022 (Figure 1). Many of these research endeavors have shown excellent results in an artificial or lab testing environment. AI has promised to revolutionize radiology and the larger field of medicine; even with the drastic increase in ML studies and products, implementation has been slower than the initial hype suggested. The year 2020 brought ML applications for radiology, and particularly screening mammography, into the public spotlight. Several papers were released within a short timeframe, one from Google’s DeepMind and one from New York University, showing that an AI application is now able to outperform a radiologist at the task of cancer identification on mammography [1,14]. While the research findings were more nuanced than the resulting inflammatory headlines the field was arguably forever changed, and both researchers and companies sought to integrate ML into real-world clinical practice. Currently, the American College of Radiology’s Data Science Institute hosts the AI central website, which aggregates a listing of FDA-cleared radiology products. At the time of article preparation, 126 radiology products are FDA-approved, 22 of which are related to breast imaging. In 2016, the first breast-imaging product was cleared with a steady increase in programs and a shift towards more diagnostic applications in subsequent years, with eight products cleared in 2021 and four products in 2022 (https://aicentral.acrdsi.org/, (accessed on 18 March 2023).

## 3. Applications for Lesion Detection beyond Mammography: Towards Breast Ultrasound

With the rapid pace of new research and products in radiology AI applications, it is important to consider the diverse array of modalities and the unique challenges they present. The transition from research to clinical practice involves not only technical hurdles but an understanding of the real-world application. One such area is ultrasound imaging. While mammography generates a uniform set of images with highly regulated positioning and imaging parameters, ultrasound images may be significantly influenced by sonographers in positioning, measurements, and imaging characteristics. Sonographers examine the target area or lesion in real time, adjusting the frequency and gain and varying the transducer’s angle, deciding on the most representative images of the region of interest. Consequently, there is a possibility for conscious or unconscious bias to result in an alteration in machine parameters that may affect the echogenicity and appearance of an image or lesion. As a result, a solid mass may unintentionally appear as a cyst or subtle nuances might be lost. When training an ultrasound model using retrospective data it is important to consider that the captured images may only represent a portion of the data used to make a final clinical decision, unintentionally biasing the algorithm. 

Automated whole breast ultrasound (ABUS) serves as an alternative to static ultrasound images or cines. This technique has gained attention for its accessibility, enabling breast cancer screening for women who may not otherwise have access to mammography. Moreover, there is evidence that supplemental screening with ultrasound could detect occult cancers, particularly in those with dense breasts [15]. ABUS generates a uniform set of images of the entire breast and saves all images to PACS. This large amount of uniformly acquired images offers an opportunity for more objective AI models compared to manual sonography where an algorithm is applied to selected ultrasound images saved at the sonographer’s discretion. A study by Hejduk et al. showed AI as having a comparable effectiveness at lesion identification on ABUS as radiologists, with an AUC of 0.91 [16]. However, a study by Brem et al. showed that the addition of ABUS to screening mammography increased cancer detection but also increased false positives [17]. By imaging the entire breast, benign lesions that may not otherwise be evaluated, such as cysts or fibroadenoma-like masses, may prompt workup, increasing patient anxiety. Further, with the potential to generate several hundred images per study ABUS is often viewed as time consuming. Algorithms may help decrease these barriers to ABUS implementation as AI-based solutions have shown promise to both increase detection and decrease reading time [15,18]. An article by Van Zelst et al. showed that CAD software provided by Qview Medical Inc. (Los Altos, CA, USA) can be used to increase the cancer detection rate of a radiologist interpreting ABUS images, and another study by Yang et al. found that both the performance and reading time of ABUS images can be improved by using AI-based software [15,19].

## 4. Obtaining More Information from the Same Images: Radiomics and Radiogenomics Advancements

Radiomics serves as a natural progression from the large volumes of uniform, three-dimensional imaging data provided by modalities such as ABUS and MRI. As an emerging field in medical imaging radiomics involves the extraction and analysis of quantitative features from medical images such as texture, shape, and intensity. Features can be correlated with variables such as patient clinical information, pathology, and molecular subtyping to create predictive or diagnostic models. When genetic information is integrated this is referred to more specifically as radiogenomics. In the analysis of breast cancer, features from imaging data are studied to improve diagnosis, prognostic modeling, and personalized treatment planning, with the aim of identifying subtle lesion changes that may be missed by visual inspection alone. For example, radiogenomics applications in ultrasound have been explored, with b-mode and vascular features correlating to several upregulated or downregulated genes [20]. Another study by Ha et al. examined postcontrast breast tumors on MRI and found that an algorithm could use imaging features to predict the genetic analysis-derived recurrence score at an AUC of 0.92, a finding that can determine the need for adjuvant chemotherapy [21]. A full review of this dynamic field is outside the scope of this broader-aiming manuscript, and dedicated discussions of radiomics and radiogenomics applications in greater depth can be found with reviews, such as Bitencourt et al., Satake et al., and Pesapane et al. [22,23,24].

## 5. Integrating Information

### 5.1. Fusing Clinical Data and Imaging

Much like the multifaceted analysis of radiomics, a mammographer’s role requires the synthesis of many factors beyond images. Mammographers rarely look at imaging in isolation; rather, they adopt a more holistic approach, considering a patient’s clinical information alongside imaging to render a diagnosis. In contrast, most commercially available software, particularly current CAD applications, analyze images without incorporating clinical information or risk models. The concept of software that integrates clinical information is rapidly gaining traction in other fields of radiology, with studies that integrated imaging and medical records doubling between 2020 and 2021 [25]. Although currently most common in the study of neurological disorders, the successful fusion of image data with non-imaging data was demonstrated in basal cell carcinoma detection by Kharazmi et al., pulmonary embolism detection by Huang et al. (2020), and prostate cancer detection on MRI images fused with the level of prostate-specific antigen by Reda et al. [26,27,28]. 

Three different strategies known as early fusion, joint fusion, and late fusion, are described in several reviews, including Huang et al. and Mohsen et al. [25,28]. The optimal approach and the most important information from images and clinical data will be determined by the specific task, with the intuitive integration of clinical information poised to enhance algorithmic performance and improve clinical care. Mohsen et al.’s. review highlights the overall success of fusion strategies, with fusion studies outperforming single-modality approaches when applied to the same tasks [25]. For instance, in a study by Reda et al. the integration of clinical information with imaging was able to achieve 94% accuracy in diagnosing prostate cancer, compared to 88% accuracy when analyzing the imaging data alone [27].

### 5.2. Information from Prior Studies

Screening mammography is intended to be performed multiple times throughout a patient’s lifetime, with at least one comparison mammogram often available for interpretation in clinical practice. In fact, one study observed that over 90% of their exams included a comparison film [29]. Most radiologists interpret mammograms within the context of comparison with a prior, allowing them to discern static lesions versus evolving changes, and increasing their confidence in the assessment. However, some evidence suggests that prior imaging may increase the callback rate due to physiologic and normal positional changes between mammograms that may appear suspicious simply due to their difference in appearance between studies. For example, a study from Yankasas et al. [29] demonstrated that when comparison mammograms were available and had an apparent change, there was an increase in the false-positive interpretation rate. However, others have shown that prior imaging allows radiologists to identify more subtle changes that may represent the early stages of cancer development, resulting in increased sensitivity. A study by Hayward et al. showed a reduced recall and increased cancer detection rate when multiple prior mammograms were available [30], and a study by Burnside et al. showed a decrease in false positives, with the detection of cancer occurring at an earlier stage [31].

Much like prior imaging may enhance the capabilities of human radiologists, incorporating prior imaging has been proposed to improve the performance of AI. Several approaches to this challenge exist, with a newer type of AI network demonstrating a high level of success in comparing two medical images to determine similarities or differences between them [32]. This network architecture is novel for its use of two parallel and identical networks to analyze the features of comparison images separately (for example, the current image and the prior image), before adding an additional component that compares between the two. Investigators have recently employed such networks in medical tasks, such as determining osteoarthritis progression on sequential knee radiographs [32] and for retinopathy grading [33]. Within breast imaging, several recent articles, including a review by Loizidou et al., and a few commercial products have emerged that specifically utilize temporal changes in medical images for better diagnosis [34]. For instance, Bai et al. compared several different types of AI networks for cancer classification, finding that the best performance was achieved with a model capable of image comparisons [35]. Using a different novel technique based on image subtraction, a study by Loizidou et al. demonstrated 99% accuracy in distinguishing masses from normal tissue in their dataset [34]. 

In addition to comparing across time points, radiologists also consider similarities and differences between the right and left breast when analyzing a study. Organ symmetry has been effectively employed in image analysis of other body regions, such as the mastoid air cells for detection of mastoiditis [18]. The integration of breast symmetry information in the academic literature is still evolving, however. A study by Shimokawa et al. demonstrated the early promise of this technique, where a network comparing the symmetry of bilateral breasts improved cancer detection compared to more traditional neural network approaches [36].

### 5.3. Challenges to Information Integration: Interoperability and Data Security

The incorporation of patient clinical information with imaging and ability to compare with prior studies are not without challenges. A significant obstacle lies in the lack of interoperability among healthcare data systems. For example, to consider a patient’s cancer history an algorithm may need permission to access the medical record, which is likely a separate application from a different company. Inconsistencies also exist in terminologies, measurement units, and data entry formats, or may need to be derived from natural language in provider notes. The scope of information an algorithm has access to also raises concerns for data privacy and security, as each new integration offers another potential source for data breaches or hacks. A healthcare data breach costs upwards of USD 6.5 million on average, making security an ethical and financial concern [37]. Addressing these challenges has the potential to improve patient care and diagnosis but will necessitate a multifaceted approach that includes standardized data formats and enhanced data management protocols.

## 6. A New Way to Derive Risk Assessment Models

Building on the integration of clinical information with imaging data is the advancement of breast cancer risk models. Enhanced risk assessment models could improve patient care by identifying vulnerable populations and promoting the targeted utilization of limited or expensive resources, such as MRI and genetic testing. Perhaps the most popular of the current risk assessment models is the Tyrer-Cuzick (TC) model, which integrates personal and family history to produce both 10-year and lifetime risk scores. In a recent multinational study, Yala et al. leveraged an algorithm to analyze both the conventional risk factors from the TC guidelines alongside information from mammographic images, finding that this algorithm was able to outperform TC in identifying both high-risk patients and individual 5-year risk [38]. 

Other studies have sought to predict risk using imaging alone. For example, Saha et al. examined features of background parenchymal enhancement on MRI as a marker for risk assessment algorithmically, finding that their multivariate model was able to identify patients who developed cancer with an AUC of 0.70 [39]. Additionally, Portnoi et al. compared a single image from screening MRIs to a logistic regression model that used more traditional risk factors for predicting 5-year risk, comparing both to the current TC models [40]. They discovered that the image-based model had the highest performance at an AUC of 0.64, while the logistic regression model and the TC model performed at AUCs of 0.56 and 0.49, respectively. These findings underscore the potential for AI-driven risk assessment models capable of integrating an increasing number of inputs to make more accurate predictions and better identify high-risk patients.

## 7. Reducing the Clinical Workload, and the Importance of Bringing Patients into the Discussion

The uses of AI in breast imaging also extend beyond diagnostic applications to address challenges in the clinical workflow. Although not currently implemented in clinical use, several studies have proposed AI for workload reduction through two primary mechanisms that involve changing the way cases are presented to the radiologist: (1) removing negative/normal cases from the worklist and (2) prioritizing abnormal cases that require prompt attention. For instance, an algorithm could analyze all screening mammography exams and automatically report those considered confidently normal, allowing radiologists to concentrate on more nuanced cases or complex diagnoses. In mammography, several groups have conducted retrospective simulations to assess workload reduction potential. Early studies demonstrated moderate benefits, with work by Rodreguez-Ruiz et al. showing a 17% reduction in studies while missing 1% of true positives, and work by Yala et al. showed no change in radiologist specificity and sensitivity while eliminating 19.3% of exams [13]. As algorithms have continued to improve, more recent research involving larger populations has shown the potential for more significant benefits. For example, a study by Shoshan et al. reported a workload reduction of 40%, with noninferior sensitivity and decreased recall rate [41]. Similarly, a large European study by Sharma et al. revealed a reading time reduction of nearly 45% while also reducing recalls [42]. By eliminating a portion of the normal exams, workload reduction algorithms have the potential to help address radiologist shortages and the potential to reduce the time patients spend waiting for anxiety-provoking results.

Despite the perceived benefit to radiologist workflows, the decision to rely on an algorithm for risk assessment or final patient diagnosis must ultimately consider several factors. Foremost among these is the level of patient comfort with reduced or absent input from a physician. The understanding of patient opinions regarding medical AI remains limited, and the landscape of AI and its integration into daily life continue to evolve. A recent meta-review by Young et al. highlighted the paucity and variability of existing studies, finding that studies examining patient attitudes were often of varying quality and were subject to selection bias [43]. Despite their overall conclusion that patients generally had a positive view of AI tools, they observed that many still prefer an element of human supervision. A breast-imaging-specific study by Lennox-Chugani [44] from England found an overall positive patient perception of using AI to read screening mammograms, with 50% of patients of screening age feeling positively and, interestingly, a slightly lower level of trust among women under screening age at 45%. The feasibility of a fully AI-based diagnosis, even for normal screening exams, should prioritize patient-centeredness and foster harmony among patients, radiologists, and administrators. Moreover, this group of stakeholders should be aware that opinions about AI-based diagnosis may change over time and maintain adaptability to ensure the best possible patient safety and comfort.

## 8. Reducing the Administrative Workload

### 8.1. Automating MQSA and General Quality Assurance

The potential of AI extends further into areas traditionally viewed as administrative, such as quality assurance tasks. Enacted in 1992, the Mammography Quality Standards Act (MQSA) mandates facilities to audit medical outcomes with the objective of establishing uniform quality standards in screening mammography. To maintain certification, MQSA requires the periodic submission of sample images to demonstrate service quality, which facilitates a comparison between an individual clinic’s performance and national-level statistics. The implementation of this audit has been shown to improve screening and diagnostic quality, with the short-interval performance feedback proving beneficial to both radiologists and technicians [45]. However, the MQSA audits necessitate the collection of a significant amount of information and the selection of appropriate images may be very time consuming, resulting in an increased administrative workload for radiologists and technologists. AI solutions have been proposed to assist with identifying images for MQSA submission, thereby reducing the administrative burden on breast imaging clinics. While there is a paucity of academic literature on this topic, several products have already entered this domain to help radiologists to streamline the process of acquiring data for EQUIP and other administrative workflows. It is noteworthy to mention that such applications for AI are relatively new, and only a limited number of products are commercially available to help radiology offices comply with MQSA and EQUIP regulations. As such, there are limited data on the actual performance and reliability of such products in clinics.

Beyond MQSA requirements, ensuring the quality of mammographic images is critical for accurate diagnoses. Poor-quality mammograms can have a significant impact on patient care, increasing radiation dose and delaying cancer detection [46]. While breast imaging phantoms can help guarantee the technical quality of mammographic equipment, human factors play a role in assuring the quality of the final image. Breast positioning has been identified as a leading cause of poor-quality images, with positioning errors contributing to misdiagnosis or missed detection of cancers [47,48]. ML-based solutions have been proposed to perform real-time quality control on acquired images, reducing the need for a technical repeat before the patient leaves the screening appointment. These types of solutions could track the performance of individual technologists, identifying areas for performance improvements, such as adequate compression or positioning, and allowing for continued and prompt feedback.

Although limited academic research has been conducted in this area, several companies have commercial products designed to automate quality-control tasks. For instance, Volpara Health reports a product that assesses factors such as position and compression on screening mammograms [49], CureMetrix, Inc has developed a product that aims to analyze a longitudinal set of studies from an institution to provide individualized quality statistics [50], and Densitas Health has a product for evaluating mammograms to flag poor-quality images and benchmark performance [51]. It is anticipated that additional products will continue to be developed that automatically ensure and maintain high-quality imaging, ultimately enhancing patient care and reducing the risk of misdiagnoses or delayed cancer detection.

### 8.2. Clinical Scheduling and Protocoling

Extending beyond the realm of imaging, AI’s efficiency can also be employed to streamline other areas of clinical operation. Several commercial AI applications have been developed to assist with clinical scheduling, aiming to optimize equipment and staff utilization. For example, algorithms have been developed to assess patient risk factors and predict the amount of time needed for a surgical case, thus enabling more accurate scheduling and utilization of operating rooms [52]. In radiology, scheduling applications may also focus on protocoling studies, such as MRIs, that have the potential to be performed differently based on the clinical scenario. Protocolling studies are essential but time-consuming and are estimated to take up to 6% of a radiologist’s time [53,54]. A study by Trivedi et al. used natural language processing (NLP), a type of machine learning, to assign contrast to musculoskeletal MRI protocols, and a study by Brown and Marotta used NLP to protocol brain MRIs, with both showing 83% accuracy [55,56]. A broader simulation study by Kalra et al. used NLP to protocol general CT and MRI studies, finding that nearly 70% of case protocols could be successfully automated [53]. As more advanced language models (such as large language models including ChatGPT) become available for study, complex clinical questions may be even better understood and translated by AI into their relevant imaging parameters. Reducing this workload could not only save time but potentially minimize interruptions and help ensure that a consistent and appropriate modality is being utilized to address the clinical question.

Appointment scheduling algorithms have also been developed, often based on the likelihood of a patient missing an appointment. This allows the schedulers to “overbook” or schedule multiple patients for the same appointment times with the expectation of attrition or cancelations. However, studies in other medical fields have found that AI scheduling may inadvertently contribute to healthcare disparities. For example, in one study, socioeconomic biases were inherent in the algorithm training data, resulting in some patients having inappropriately longer wait times [57]. ML applications are ultimately a reflection of their training data, and understanding that ML applications may perpetuate human biases is important to ensure that vulnerable populations are receiving equitable care. Multiple studies have underscored the imperative for a cautious and ethical approach towards creating AI models, with a clear focus on enhancing data diversity to ensure equitable health outcomes for all populations. For example, Mema and McGinty discussed the potential for AI to reduce health disparities in breast cancer care and highlight the need for active clinician engagement to reduce biases, Agarwal et al. discussed bias sources and proposed mitigation strategies in AI for healthcare, and Halamka et al. discussed discrimination relating to surgical care and proposed ways AI may help [58,59,60]. 

## 9. The Limitations of AI, and Carrying Its Promises into the Future

AI has already begun to revolutionize medical imaging; however, like with any emerging technology it is not without limitations or growing pains. One present concern is the quality and standardization of data used to train AI algorithms. Although larger public datasets are becoming more accessible for applications, such as cancer detection on mammography, many companies and researchers must create their own datasets for applications, such as scheduling or comparisons with prior studies. Typically, companies select their own set of testing data and submit their results to the FDA for approval. Without high-quality training data, an algorithm may face issues with generalizability outside its developing institution or may perpetuate unintentional biases present in its training set. Further, quality tools for benchmarking performance remain underdeveloped in medical AI applications, requiring a consumer to rely on testing results provided by a company or researcher without the ability to directly compare the results between studies or companies on the same datasets. 

Careful consideration must be paid to the role of an AI application within a clinical workflow, evaluating whether it slows the workflow (e.g., waiting times for an image to be processed) or increases analysis time for radiologists, particularly at the implementation and integration phases. These factors are particularly important when considering the IT infrastructure of an organization, which may vary in age or be costly and difficult to upgrade and integrate into. The interpretability of AI-based decision making is another important consideration, as healthcare providers may be liable for the end result and may not trust an AI tool if its workings are opaque or difficult to understand. Finally, updates and maintenance of an AI algorithm must be considered to ensure optimal performance over time, accounting for potential changes due to software updates or population drift. 

In the future, AI applications hold the potential to address many challenges faced in the clinical practice of medical imaging. While lesion identification remains an essential task in breast imaging, particularly in the context of a shortage of mammographers, AI has the potential to streamline aspects of the breast radiologist workflow and improve patient care. For example, with the advent of advanced language processing and generation tools such as ChatGPT, AI could assist in evaluating years of patient histories and summarizing them meaningfully. Patient data could also be integrated from multiple sources, including electronic health records, lab results, and imaging studies, providing physicians with a more comprehensive view of a patient’s health and enabling clinicians to make more informed decisions. AI could also be utilized to automatically generate draft reports, reducing the time needed for documentation. These AI applications have the potential to significantly enhance the efficiency and accuracy of medical imaging, ultimately leading to more efficient and effective radiologists and improved patient care. 

## Data Availability

No new data were created or analyzed in this study. Data sharing is not applicable to this article.
